# Immune Receptors Involved in *Streptococcus suis* Recognition by Dendritic Cells

**DOI:** 10.1371/journal.pone.0044746

**Published:** 2012-09-12

**Authors:** Marie-Pier Lecours, Mariela Segura, Nahuel Fittipaldi, Serge Rivest, Marcelo Gottschalk

**Affiliations:** 1 Department of Pathology and Microbiology, Faculty of Veterinary Medicine, Université de Montréal, St-Hyacinthe, Québec, Canada; 2 Department of Pathology and Genomic Medicine, The Methodist Hospital System, Houston, Texas, United States of America; 3 Laboratory of Endocrinology and Genomics, Centre Hospitalier de l'Université Laval, St-Foy, Québec, Canada; Mayo Clinic, United States of America

## Abstract

*Streptococcus suis* is an important swine pathogen and an emerging zoonotic agent of septicemia and meningitis. Knowledge on host immune responses towards *S. suis*, and strategies used by this pathogen for subversion of these responses is scarce. The objective of this study was to identify the immune receptors involved in *S. suis* recognition by dendritic cells (DCs). Production of cytokines and expression of co-stimulatory molecules by DCs were shown to strongly rely on MyD88-dependent signaling pathways, suggesting that DCs recognize *S. suis* and become activated mostly through Toll-like receptor (TLR) signaling. Supporting this fact, TLR2^−/−^ DCs were severely impaired in the release of several cytokines and the surface expression of CD86 and MHC-II. The release of IL-12p70 and CXC10, and the expression of CD40 were found to depend on signaling by both TLR2 and TLR9. The release of IL-23 and CXCL1 were partially dependent on NOD2. Finally, despite the fact that MyD88 signaling was crucial for DC activation and maturation, MyD88-dependent pathways were not implicated in *S. suis* internalization by DCs. This first study on receptors involved in DC activation by *S. suis* suggests a major involvement of MyD88 signaling pathways, mainly (but not exclusively) through TLR2. A multimodal recognition involving a combination of different receptors seems essential for DC effective response to *S. suis*.

## Introduction

*Streptococcus suis* serotype 2 is a major swine pathogen mainly associated with meningitis, although other systemic infections have been described [1], [2]. *S. suis* is now emerging as a threat to human health, especially in Asian countries where it has recently been identified as the leading cause of adult meningitis in Vietnam, the second in Thailand, and the third in Hong Kong [2]. Moreover, two important human outbreaks of streptococcal toxic shock-like syndrome (STSLS) due to *S. suis* occurred in China during the last years with a fatality rate near 20% [2].

Several virulence factors have been proposed to be involved in the pathogenesis of the infection. The most important among them is the capsular polysaccharide (CPS), which confers antiphagocytic properties to the pathogen [3]. In addition, the bacterial cell wall and modifications of its components, such as the N-deacetylation of peptidoglycan (PG) and the D-alanylation of lipoteichoic acids (LTA), were shown to also contribute to the virulence of *S. suis* [4]–[6]. Other virulence factors have also been proposed [3], [7]. Among them, an hemolysin (suilysin), although not considered as a critical virulence factor [8] and being absent in many virulent strains [9], has been shown to play a certain role in *in vitro* interactions between *S. suis* and different host cells [1], [7], [10], [11].

As evidenced by human *S. suis* outbreaks of STSLS as well as by septic shock cases in Europe and Asia, an important release of pro-inflammatory mediators is thought to take place during *S. suis* systemic infections [2]. In fact, *S. suis* is able to induce *in vitro* production of different pro-inflammatory cytokines and chemokines by porcine, murine, and human cells; and *in vivo* upregulation of inflammatory mediators in affected humans as well as in experimental mouse models of infection [12]–[14].

DCs are powerful antigen-presenting cells that initiate immune responses against pathogens; they capture and process antigens, and then undergo a maturation process characterized by the production of cytokines and upregulation of co-stimulatory molecules. Then, DCs migrate to adjacent lymphoid organs where they activate T cells [15]. Recognition of pathogen-associated molecular patterns (PAMPs) by DCs is mediated by pattern-recognition receptors (PRRs), including the Toll-like receptor (TLR) and nucleotide-binding oligomerization domain (NOD)-like receptor (NLR) families [16]. TLR2 is reported to be specialized for the recognition of lipoproteins by generally forming a heterodimer with TLR1 or TLR6 [17], [18]. Although TLR4 has been shown as important for the recognition of lipopolysaccharide (LPS) [17], it has also been reported to recognize the pneumolysin, a suilysin-related toxin produced by *Streptococcus pneumoniae* [19], [20]. TLR9 is an intracellular receptor involved in the recognition of bacteria-derived DNA [17]. At least two well-characterized NLRs, that is, NOD1 and NOD2, recognize the structures of bacterial PGs, g-D-glutamyl-meso-diaminopimelic acid and muramyl dipeptide, respectively [18]. Another group of NLRs participates in the formation of a large multiprotein complex called the inflammasome, whose assembly leads to the activation of caspase 1-mediated innate immune responses [18].

Interactions between TLRs and NODs with their ligands initiate an intracellular signaling cascade that induces the secretion of several pro-inflammatory cytokines and the expression of co-stimulatory cell-surface molecules through the activation of transcription factors including NF-κB [18]. Signaling occurs through association of TLRs with several adaptor molecules, such as the myeloid differentiation factor 88 (MyD88) [18]. Pathogens can, however, hijack the TLR signaling to evade recognition and elimination by the immune system [21]. TLRs and NODs can synergistically activate proinflammatory cytokine production [16].

Bone marrow-derived DCs (bmDCs) have been shown to be a valid and interesting model to study the host immune response during *S. suis* infection [10]. Using this model, it has been shown that *S. suis* uses an arsenal of different virulence factors to modulate DC functions, particularly cytokine release and complement-dependent opsono-phagocytosis [10], [22]. As such, we hypothesize that *S. suis* activates cells through multiple receptors. In the present study, we used bmDCs to evaluate the importance of specific immune receptors in the recognition of *S. suis* serotype 2.

**Table 1:** Bacterial strains and plasmids used in this study.

Strains/Plasmids	General characteristics	Source/Reference
***Escherichia coli***		
TOP10	F- *mcr*A Δ(*mrr-hsd*RMS-*mcr*BC)φ80*lac*ZΔM15 Δ*lac*X74 *rec*A1 *ara*D139 Δ(*ara-leu*) 7697 *gal*U *gal*K *rps*L (StrR)*end*A1 *nup*G	Invitrogen
***Streptococcus suis***		
31533	Wild type, highly virulent strain isolated from a pig with meningitis.Serotype 2.	[23]
B218	Non-encapsulated mutant strain derived from strain 31533.	[13]
Δ*dltA*/Δ*pgdA*	Mutant deficient for the D-alanylation of LTA and the N-deacetylation ofPG. Derived from strain 31533.	This work
Δ*sly*	Mutant deficient for the production of suilysin. Derived from strain 31533.	[8]
**Plasmids**		
pCR2.1	Ap^r^, Km^r^, *oriR*(f1) MCS *oriR* (ColE1)	Invitrogen
pSET5s	Thermosensitive vector for allelic replacement is *S. suis*. Replicationfunctions of pG+host3, MCS *oriR* pUC19 *lacZ* Sp^R^	[25]
P5ΔdltA	pSET5s carrying the construct for *dltA* allelic replacement	This work

LTA; lipoteichoic acid, PG; peptidoglycan.

## Materials and Methods

### Ethics Statement

All experiments involving mice were conducted in accordance with the guidelines and policies of the Canadian Council on Animal Care and the principles set forth in the Guide for the Care and Use of Laboratory Animals by the Animal Welfare Committee of the Université de Montréal (Comité d’éthique de l’utilisation des animaux (CÉUA)). The protocols and procedures were approved by the Ethics Committee (CÉUA).

**Table 2:** Oligonucleotide primers used in this study for construction of in-frame deletion mutants.

Primer name	Sequence (5′ –3′)^a^
ID.1_dlTA_left_FWD	CACTCATTACAACTCTCGCAG
ID.2_dlTA_left_REV	TCCAAACTATCAATATGGGCTG
ID.3_dlTA_right_FWD	GCTTATGTTGTCCCTAAAGCAG
ID.4_dlTA_left_REV	GCCCATCAAGAGCATATTTAGC
ID.5_dlTA_left_FWD	AGACCTCACATTTTTTGCG
ID.6_dlTA_left_REV	GTCAAAGGAAGACTGTCTCGGTAGTCAGGATTTTCTGTCG
ID.7_dlTA_right_FWD	CGACAGAAAATCCTGACTACCGAGACAGTCTTCCTTTGAC
ID.8_dlTA_right_REV	TCAATCACCATTCCGACCG

a Oligonucleotide primers were from Invitrogen.

### Bacterial Strains, Plasmids and Growth Conditions

Bacterial strains and plasmids used are described in Table 1. *S. suis* strains were grown on Todd-Hewitt broth (THB) or agar (Becton Dickinson, Mississauga, ON, Canada) or on sheep blood agar plates at 37°C. *Escherichia coli* was grown on Luria-Bertani broth or agar (Becton Dickinson). When needed, antibiotics (Sigma, Oakville, ON, Canada) were added to the culture media at the following concentrations: for *E. coli*, kanamycin and spectinomycin at 50 µg/ml; for *S. suis*, spectinomycin at 100 µg/ml. To perform *S. suis*-DCs interaction studies, bacteria suspensions were prepared as previously described [10] and appropriately diluted in complete cell culture medium for the experiments. The number of CFU/ml in the final suspension was determined by plating samples onto THB agar using Autoplate® 4000 Automated Spiral Plater (Spiral Biotech, Norwood, MA).

**Figure 1:**
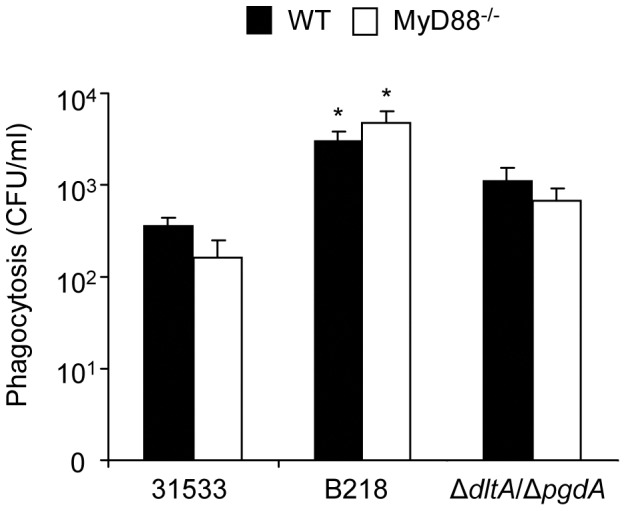
Effect of MyD88 deficiency on the capacity of DCs to internalize *S. suis*. Bacteria (10^6^ CFU/ml) were pre-opsonized with 20% complete normal mouse serum for 30 min prior to incubation with DCs (10^6^ cells/ml). Phagocytosis was left to proceed for 2 h before antibiotics were directly added into the wells for 1 h to kill extracellular bacteria. Viable intracellular bacteria were determined by quantitative plating of serial dilutions of the lysates onto THB agar. * P<0.05 denotes values that are significantly different from those obtained with the *S. suis* parental strain 31533.

### Construction of the Knockout Vector for Gene Replacement and Generation of *S. suis* Δ*dltaA*/Δ*pgdA* double Knockout

Δ*dltaA* and Δ*pgdA* mutants were produced and characterized in our laboratory in the past [4], [5]. In order to evaluate a combined effect of LTA and PG modifications, a Δ*dltaA*/Δ*pgdA* double mutant was generated. Briefly, genomic DNA from parent strain 31533 was prepared using InstaGene Matrix (BioRad Laboratories, Mississauga, ON, Canada). Then, a 1407 bp, precise, in-frame deletion of the *dltA* gene was constructed by using splicing-by-overlap-extension PCR [24] and the primers (Invitrogen, Burlington, ON, Canada) listed in Table 2. The PCR-generated Δ*dltA* deletion allele was subsequently cloned into plasmid pCR2.1 (Invitrogen), extracted with BamHI and PstI and recloned into the same sites of the thermosensitive *E. coli-S. suis* shuttle plasmid pSET5s, which carries the chloramphenicol resistance gene *cat* [25]. The resulting knock-out vector was named p5ΔdltaA. Restriction enzymes and DNA-modifying enzymes (TaKaRa Bio, Otsu, Shiga, Japan) were used according to the manufacturers’ recommendations. PCR reactions were carried out with the iProof proofreading DNA polymerase (BioRad). Minipreparations of recombinant plasmids and transformation of *E. coli* were performed by standard procedures [26]. To obtain the double mutant, knock-out vector p5ΔdltaA was electroporated into the previously generated *S. suis* Δ*pgdA* mutant strain. Procedures for isolation of mutants were those described previously [27]. Successful allelic replacement of the *dltA* gene in the Δ*pgdA* strain was confirmed by PCR and DNA sequencing analysis using an ABI 3730xl automated DNA sequence and the ABI PRISM dye terminator cycle version 3.1 (Applied Biosystems, Carlsbad, CA).

**Figure 2:**
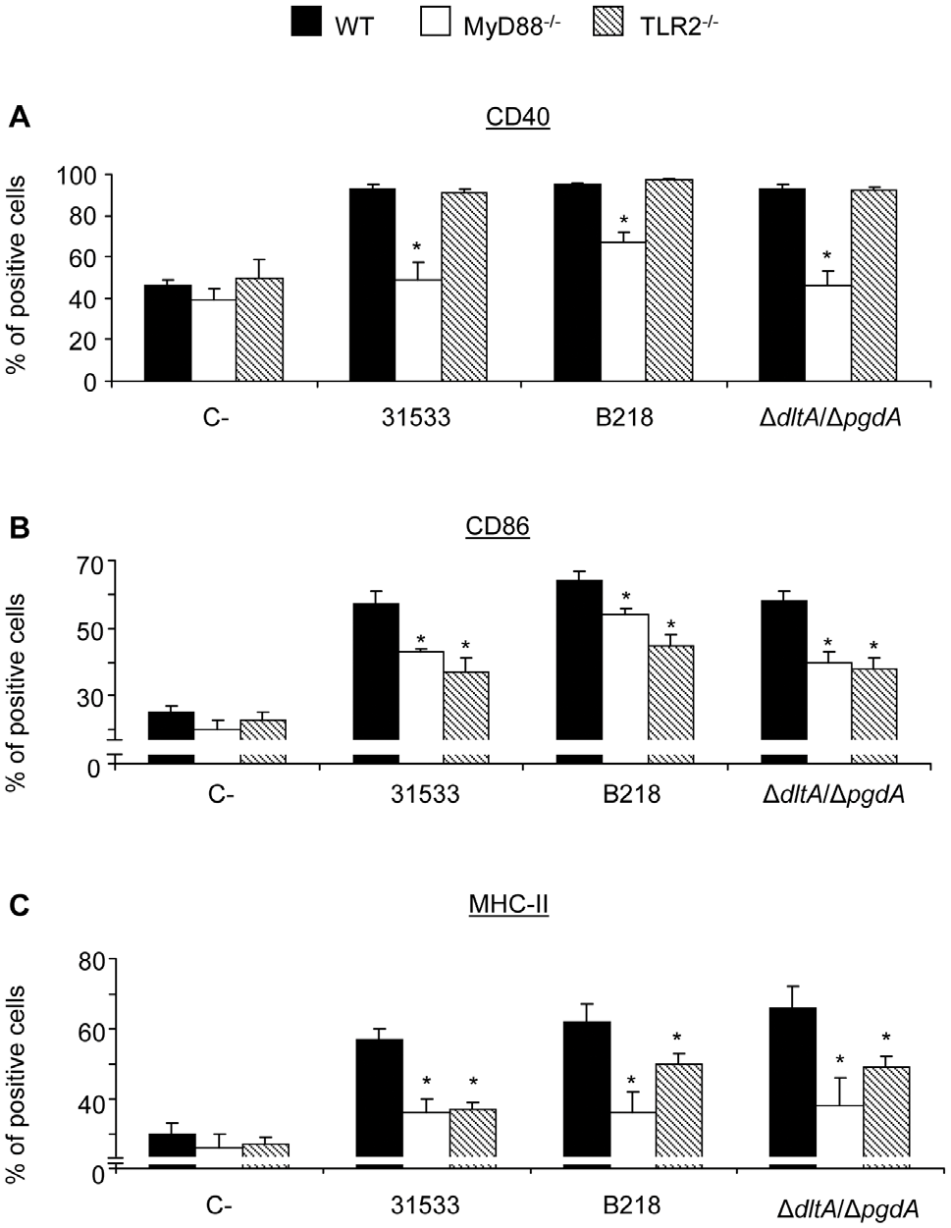
Surface expression of co-stimulatory molecules by DCs in response to *S. suis*. WT, MyD88^−/−^, and TLR2^−/−^ DCs (10^6^ cells/ml) were stimulated with S. *suis* (10^6^ CFU/ml) for 16 h. Non-stimulated cells served as negative control (C−). (A) Percentage of CD40 positive cells. (B) Percentage of CD86^high^ positive cells. (C) Percentage of MHC-II^high^ positive cells. Twenty thousand gated events were acquired per sample. Quadrants were drawn based on FITC- and PE-control stains and were plotted on logarithmic scales. CD40, CD86 and MHC-II histograms were obtained by gating cells based on positive CD11c staining. * P<0.05 denotes values that are significantly lower than those obtained with WT DCs.

### Generation of Mouse Bone Marrow-derived Dendritic Cells (bmDCs)

Six to eight week-old mice originated from Jackson Laboratory (Bar Harbor, ME, USA), including wild type (WT) C57BL/6, MyD88^−/−^ (B6.129P2-*Myd88^tm1Defr^*/J), TLR2^−/−^ (B6.129-*Tlr2* ^tmlKir^/J), TLR4^−/−^ (B6.B10ScN-*Tlr4^lps-del^*/JthJ) and NOD2^−/−^(B6.129S1-*Nod2^tm1Flv^*/J) mice were used. BmDCs were produced according to a technique previously described [10]. Briefly, on day 0, bone marrow was removed from femurs and tibiae. After red blood cell lysis, total bone marrow cells (2.5×10^5^ cells/ml) were cultured in complete medium consisting of RPMI 1640 supplemented with 5% heat-inactivated fetal bovine serum, 10 mM HEPES, 20 µg/ml gentamycin, 100 U/ml penicillin-streptomycin, 2 mM L-glutamine and 50 µM 2-mercaptoethanol. All reagents were from Gibco (Burlington, ON, Canada). Complete medium was complemented with 20% GM-CSF from a mouse GM-CSF-transfected cell line (Ag8653) as a source of GM-CSF [28]. Cells were cultured for 7 days at 37°C in a 5% CO_2_ incubator and were fed on days 3 and 5. On day 7, clusters were harvested and subcultured overnight to remove adherent cells. Non-adherent cells were collected on day 8, washed, and used as immature DCs for the studies. Cell purity was routinely 86–90% CD11c^+^ cells as determined by FACS analysis and as previously reported [10].

**Figure 3:**
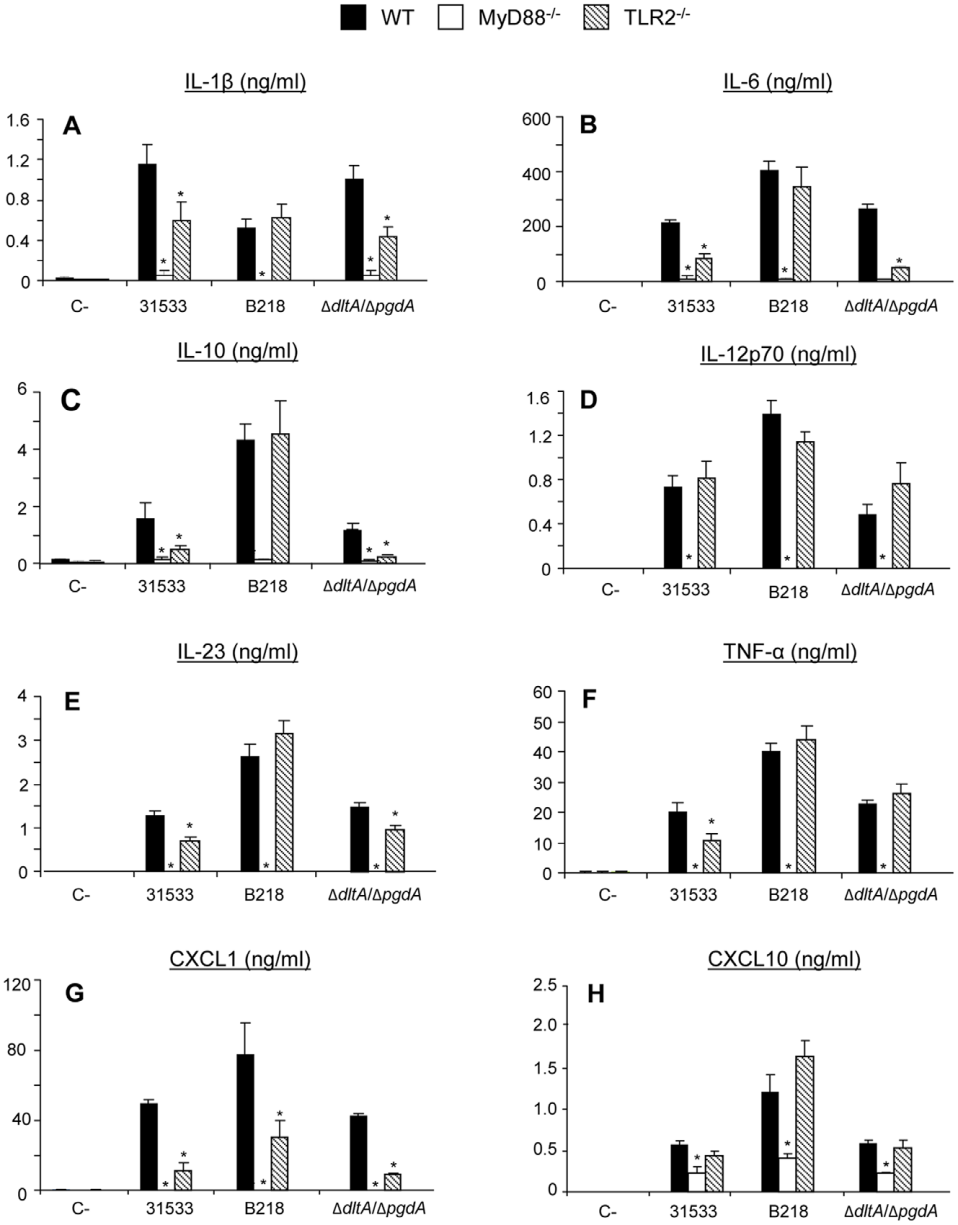
Cytokine production by DCs in response to *S. suis*. WT, MyD88^−/−^, and TLR2^−/−^ DCs (10^6^ cells/ml) were stimulated by different *S. suis* strains (10^6^ CFU/ml) for 16 h. Non-stimulated cells served as negative control (C−). Sample dilutions giving optical density readings in the linear portion of the ELISA standard curves were used to quantify cytokine levels. * P<0.05 denotes values that are significantly lower than those obtained with WT DCs.

**Figure 4:**
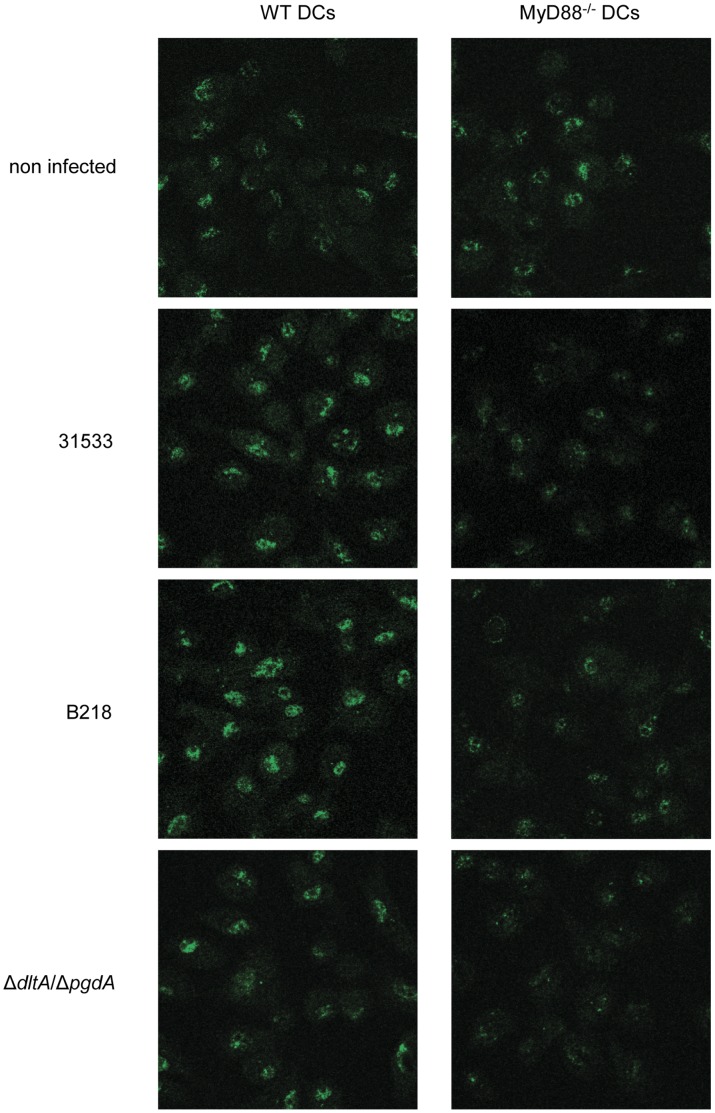
Effect of MyD88 deficiency on NF-κB expression by *S. suis* infected-DCs. WT DCs or MyD88^−/−^ DCs were incubated with the parental strain 31533, the non-encapsulated mutant B218 or the cell wall mutant Δ*dltA*/Δ*pgdA* strain (10^6^ CFU/ml). After a bacterial-cell contact of 8 h, cells were fixed and labeled with an antibody specific for NF-κB p65 (Alexa-Fluor 488, green) and analyzed by confocal microscopy.

### Phagocytosis Assay

Bacteria were pre-opsonized using 20% complete normal mouse serum in PBS for 30 min at 37°C with agitation, as previously described [10]. DCs (10^6^ cells/ml) were infected with pre-opsonized *S. suis* strains (31533, B218 and Δ*dltA*/Δ*pgdA* at a MOI: 1). Phagocytosis was left to proceed for 2 h at 37°C with 5% CO_2_. MOI and assay conditions were chosen based on previous studies on the kinetics of *S. suis* phagocytosis by DCs [22]. After incubation, penicillin G (5 µg/ml) and gentamycin (100 µg/ml) (both from Sigma) were directly added into the wells for 1 h to kill extracellular bacteria. Supernatant controls were taken in every test to confirm that extracellular bacteria were efficiently killed by the antibiotics. After antibiotic treatment, cells were washed 3 times, and sterile water was added to lyse the cells. To ensure complete cell lysis, cells were disrupted by scraping the bottom of the well and by vigorous pipetting. Each test was repeated at least four times in independent experiments, and the number of CFU recovered per well (mean number ± SEM) was determined by viable intracellular bacterial counting as described above.

**Figure 5:**
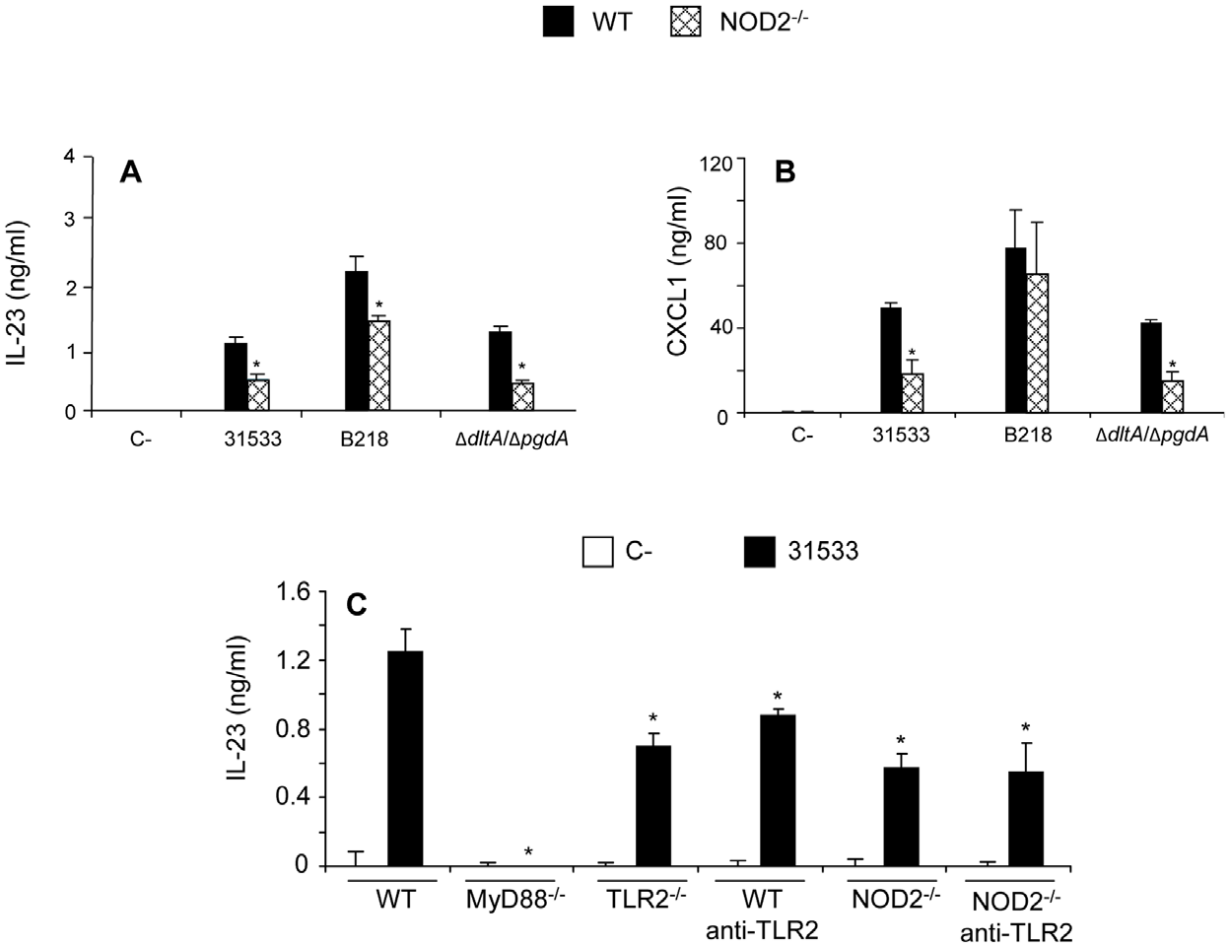
Role of NOD2 receptor in cytokine production by *S. suis*-stimulated DCs. WT and NOD2^−/−^ DCs (10^6^ cells/ml) were stimulated by different *S. suis* strains for 16 h. Non-stimulated cells served as negative control (C-). The production of IL-23 (A) and CXCL1 (B) were measured. (C) WT DCs and NOD2^−/−^ DCs (10^6^ cells/ml) pre-treated or not with a neutralizing anti-TLR2 antibody (clone T2.5; 15 µg/ml) were stimulated by *S. suis* parental strain 31533 (10^6^ CFU/ml) for 16 h, and the release of IL-23 was analyzed by ELISA. For comparative purposes, MyD88^−/−^ DCs and TLR2^−/−^ DCs were also included. Sample dilutions giving optical density readings in the linear portion of the ELISA standard curves were used to quantify cytokine levels. * P<0.05 denotes values that are significantly lower than those obtained with WT DCs.

### In vitro DC Stimulation Assay

DCs were resuspended at 10^6^ cells/ml in complete medium supplemented with 5% GM-CSF supernatant and stimulated with different strains of *S. suis* (10^6 ^CFU/ml; initial MOI: 1). Conditions used were based on those already published [10]. Bacterial strains were pre-opsonized using 20% complete normal mouse serum as described above. At different time intervals, supernatants were collected for cytokine quantification by ELISA and cells were harvested for analysis of co-stimulatory molecule expression by FACS. Non-stimulated cells served as negative control. Lactate dehydrogenase (LDH) release measurement assay was used to confirm absence of cytotoxicity in bacterial-bmDC cultures (Promega CytoTox96, Promega Corporation, Madison, WI, USA), as previously described [14].

**Figure 6:**
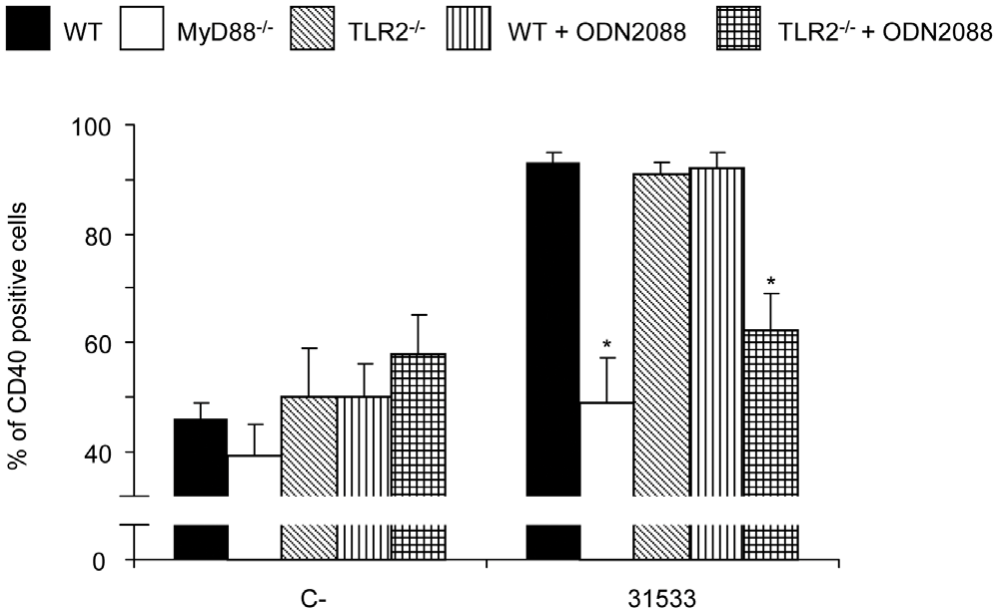
CD40 expression by DCs in response to *S. suis* depends on both TLR2 and TLR9. WT DCs and TLR2^−/−^ DCs (10^6^ cells/ml) pre-treated or not with an antagonist for TLR9 (ODN2088; 5 µM), were stimulated with *S. suis* parental strain 31533 (10^6^ CFU/ml) for 16 h. Non-stimulated cells served as negative control (C-). For comparative purposes, MyD88^−/−^ DCs were also included. Twenty thousand gated events were acquired per sample. Quadrants were drawn based on FITC- and PE-control stains and were plotted on logarithmic scales. Histograms were obtained by gating cells based on positive CD11c staining. * P<0.05 denotes values that are significantly lower than those obtained with WT DCs.

For inhibition of TLR9, DCs were pre-treated with ODN2088 (5 µM) (Invivogen, Burlington, ON, Canada) for 1 h prior to infection with *S. suis*. The TLR9 activator ODN1826 (1 µM) (Invivogen) was used as a positive control to stimulate bmDCs through TLR9 [29]. For neutralization of TLR2, bmDCs were pre-treated for 1 h with 15 µg/ml of anti-TLR2 (clone T2.5, Hycult biotechnology, Plymouth, PA). PAM(3)CSK(4) (TLR1/2 ligand, final concentration of 500 ng/ml), FSL-1 (TLR2/6 ligand, final concentration of 500 ng/ml) and ultra pure LPS (TLR4 ligand, final concentration of 1 µg/ml) were used as positive controls (Invivogen) (data not shown).

**Figure 7:**
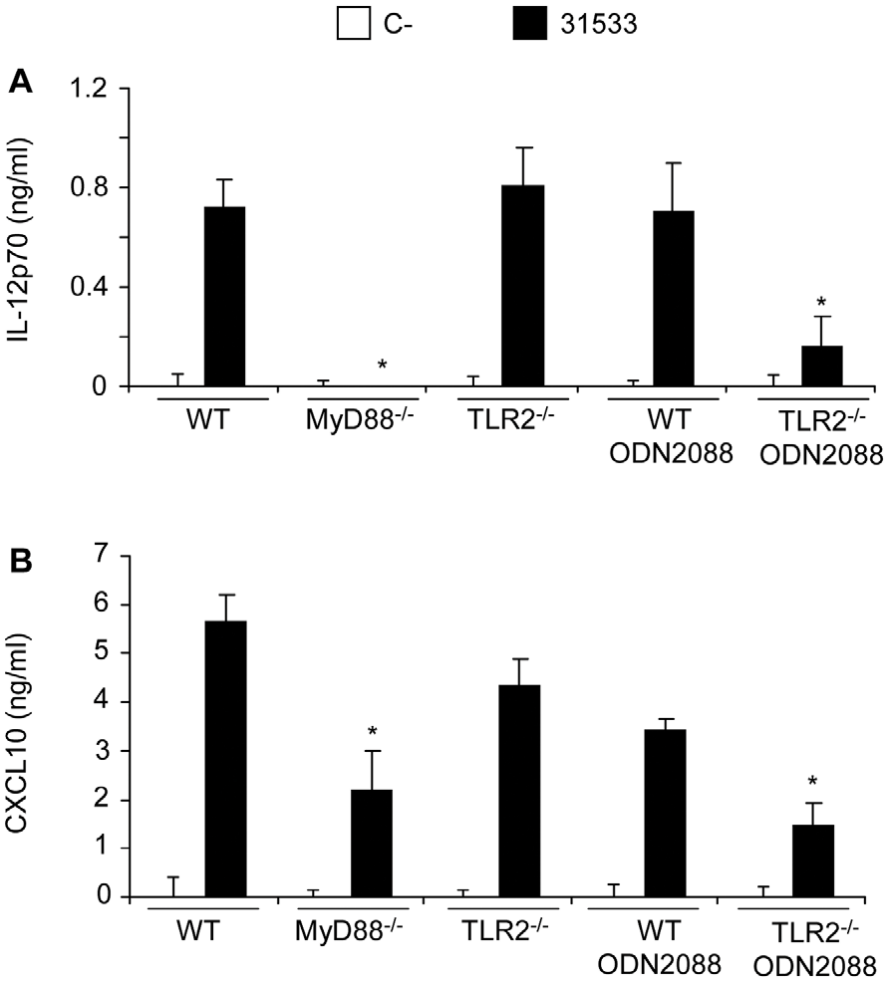
Role of TLR2 and TLR9 in IL-12p70 and CXCL10 production by *S. suis*-stimulated DCs. WT DCs and TLR2^−/−^ DCs (10^6^ cells/ml) pre-treated or not with an antagonist for TLR9 (ODN2088; 5 µM), were stimulated with *S. suis* parental strain 31533 (10^6^ CFU/ml) for 16 h, and the release of IL-12p70 (A) and CXCL10 (B) were analyzed by ELISA. Non-stimulated cells served as negative control (C-). For comparative purposes, MyD88^−/−^ DCs were also included. Sample dilutions giving optical density readings in the linear portion of the ELISA standard curves were used to quantify cytokine levels. * P<0.05 denotes values that are significantly lower than those obtained with WT DCs.

### Cytokine Quantification by ELISA

Levels of IL-1β, IL-6, IL-10, IL-12p70, IL-23p19, TNF-α, CXCL1 and CXCL10 in cell culture supernatants were measured by sandwich ELISA using pair-matched antibodies from R&D Systems (Minneapolis, MN) or eBioscience (San Diego, CA), according to the manufacturer’s recommendations.

**Figure 8:**
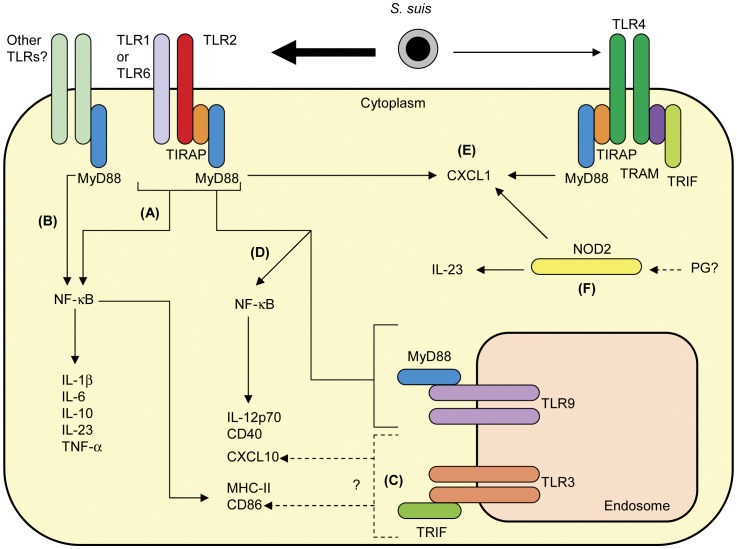
Proposed model of *S. suis* recognition by DCs. (A) The release of IL-1β, IL-6, IL-10, IL-23 and TNF-α is TLR2-dependent. TLR2 is also involved in the surface expression of MHC-II and CD86. (B) Other TLRs would also be implicated in the release of IL-1β, IL-6, IL-10, IL-23 and TNF-α. (C) TLR3 might be involved in the MyD88-independent production of CXCL10 and expression of CD86. (D) Collaboration between TLR2 and TLR9 is involved in the production of IL-12p70 and CXCL10 and the expression of CD40. (E) Collaboration among TLR2 and NOD2, with a minor contribution of TLR4, is involved in the release of CXCL1. (F) NOD2 also contributes to the release of IL-23. Recognition of *S. suis* peptidoglycan (PG) might be involved in NOD2 activation.

### FACS Analysis

For cell surface staining, 10^6^ cells were washed and treated for 30 min on ice with FcR-blocking reagent (FcγIII/II Rc Ab, BD PharMingen, Mississauga, ON, Canada) in sorting buffer (PBS-1% fetal bovine serum). Blocked cells were then incubated with FITC-labeled anti-mouse CD11c mAb clone HL3 (BD PharMingen) for 1 h on ice followed by washing and staining for 1 h with a PE-labeled monoclonal antibody against the following surface molecules: CD86 (clone GL1), CD40 (clone 3/23), and MHC class II (A_β_^b^; clone AF6–120.1) from BD PharMingen. After washing, cells were resuspended in sorting buffer for FACS analysis. Flow cytometry was performed using a FACSCalibur instrument (BD Biosciences, Mississauga, ON, Canada). Twenty thousand gated events were acquired per sample and data analysis was performed using CellQuest software. Quadrants were drawn based on FITC- and PE-control and isotype control stains and were plotted on logarithmic scales.

### Confocal Microscopy

For immunofluorescence studies, DCs (10^6^ cells) were placed on coverslips and infected with different strains of *S. suis* (10^6^ CFU/ml, MOI: 1). After 8 h of bacteria-cell contact, coverslips were washed with PBS to remove non-associated bacteria, and cells fixed with methanol/acetone (80∶20) for 20 min at −20°C, and then washed and blocked for 10 min. Coverslips were incubated for 1 h with rabbit anti-NF-κB p65 (Ser 276) antibody (Santa Cruz Biotechnology, Santa Cruz, CA). After washing, coverslips were incubated with the secondary antibodies Alexa-Fluor 488 goat anti-rabbit IgG (Invitrogen) for 30 min, washed and mounted on glass slides with moviol containing DABCO and DAPI to stain the nuclei. Samples were observed with an IX-80 confocal microscope integrated into the FV-1000 imagery system and analysed using the fluoview software (Olympus Canada, Richmond Hill, ON, Canada).

### Statistical Analysis

All data are expressed as mean ± SEM. Data were analyzed for significance using ANOVA analysis. A P value <0.05 was used as a threshold for significance. All experiments were repeated at least three times.

## Results

### Internalization of *S. suis* is Independent on TLR Signalization

It has previously been reported that TLRs may be implicated as receptors for bacterial phagocytosis [30]. In order to globally evaluate their implication, we investigated if DC deficiency in MyD88 expression would affect the internalization of *S. suis*. The number of bacteria internalized by MyD88^−/−^ DCs was not significantly different from those obtained with WT DCs for the parental as well as mutant strains (Figure 1). Hence, deficiency in MyD88 signaling does not seem to play a major role in the ability of DCs to internalize *S. suis*. As expected, the non-encapsulated mutant strain was significantly more internalized by DCs than the parental strain 31533 (Figure 1).

### Role of Different Receptors for DC Maturation in Response to *S. suis* Infection

The role of different receptors and signaling pathways in the maturation of DCs by *S. suis* was evaluated by studying the expression of the co-stimulatory molecules CD40, CD86 and MHC-II on DCs from WT or knock-out mice. Compared to control cells, *S. suis*-stimulated WT DCs showed higher surface expression levels of CD40, CD86 and MHC-II mice in terms of the percentage of cells expressing these markers (Figure 2 and Figure S1) as well as in MFI levels (data not shown). As expected [10], two well segregated sub-populations, a CD86^high^/MHC-II^high^ subset and a CD86^low^/MHC-II^low^ subset, are constantly observed among the CD11c^+^ DC population following *S. suis* infection. As shown in Figure 2, the expression of CD40 and MHC-II was significantly reduced in MyD88^−/−^ DCs following *S. suis* infection, reaching levels similar to those observed in non-activated control cells (Figure 2A, C). Interestingly, the expression of CD86 in MyD88^−/−^ DCs after *S. suis* activation was also significantly reduced but still higher than basal levels, suggesting a partial requirement of MyD88 signaling for CD86 expression (Figure 2B). Therefore, DC expression of surface molecules in response to *S. suis* occurs mainly but not exclusively through a MyD88-dependent pathway.

These results suggested that signaling through TLRs is the main pathway by which DCs sense *S. suis* and become activated. Hence, we investigated the participation of TLR2 in DC maturation following stimulation with *S. suis*. For all strains tested, no significant differences between the WT DCs and the TLR2^−/−^ DCs were observed for the expression of CD40, suggesting that the expression of this marker is TLR2-independent (Figure 2A). However, analysis of number of cells expressing the CD86^high^ and MHC-II^high^ subsets, revealed that the expression of these molecules were significantly reduced in TLR2^−/−^ DCs infected with *S. suis* (Figure 2B, C). The CPS and cell wall modifications do not seem to play an important role in modulating co-stimulatory molecule expression through TLR2/MyD88 signaling as both mutant strains behaved similarly to the parental strain (Figure 2). No differences were observed between WT DCs and either TLR4^−/−^ or NOD2^−/−^ DCs in the ability to up-regulate expression of the above mentioned co-stimulatory molecules following stimulation with *S. suis*, neither in terms of percentage of cells expressing these molecules or in terms of MFI (data not shown).

### Role of Different Receptors on DC Activation in Response to *S. suis* Infection

The contribution of different receptors in DC cytokine production following stimulation with *S. suis* was investigated. DCs were incubated with different *S. suis* strains for 16 h. Optimal assay conditions were chosen based on previous results [10] and preliminary studies on the kinetics of cytokine release by DCs in response to *S. suis* (data not shown). The levels of the pro-inflammatory cytokines IL-1β, IL-6 and TNF-α, the Th1-driving cytokines IL-12p70 and IL-23, the regulatory cytokine IL-10, and the chemokines CXCL1 and CXCL10 in the supernatants of *S. suis*-infected DCs were measured. Production of these mediators was either completely abrogated or dramatically impaired in MyD88^−/−^ DCs for all strains tested (Figure 3). In addition, the nuclear expression of NF-κB was significantly reduced in *S. suis*-stimulated MyD88^−/−^ DCs for all strains tested, confirming participation of MyD88 signaling pathways in DC activation and maturation in response to *S. suis* (Figure 4).

The involvement of TLR2 in DC cytokine production following stimulation with *S. suis* was also investigated using TLR2^−/−^ DCs. The release of IL-1β, IL-6, IL-10, IL-23, TNF-α and CXCL1 was significantly reduced in TLR2^−/−^ DCs infected with *S. suis* parental strain (Figure 3A, B, C, E, F and G). On the other hand, the release of IL-12p70 and CXCL10 was found to be TLR2-independent (Figure 3D and H). Conversely to the parental strain, the non-encapsulated strain B218 maintained its capacity of inducing most cytokines in TLR2^−/−^ DCs, with the exception of CXCL1 (Figure 3G), indicating that high surface expression levels of cell wall components (normally hidden by the CPS) are able to activate cells through other TLRs. The cell wall mutant strain Δ*dltA*/Δ*pgdA* behaved exactly as the parental strain 31533, except for the release of TNF-α, which was found to be TLR2-independent (Figure 3F). Overall these results indicate that the release of most cytokines by *S. suis*-stimulated DCs involves TLR2. However, the fact that the inhibition of cytokine release in TLR2^−/−^ DCs was still significantly different (P<0.05) from the inhibition observed with MyD88^−/−^ DCs, suggests that TLR2-independent pathways would also be involved in DC activation by *S. suis*.

Of all cytokines and chemokines tested, only CXCL1 was reduced following TLR4^−/−^ DC stimulation with *S. suis* parental strain 31533 and its Δ*dltA*/Δ*pgdA* mutant (Figure S2). Since TLR4 is known to mediate the recognition of *S. pneumoniae* pneumolysin, a suilysin-related toxin, we evaluated the release of CXCL1 by TLR4^−/−^ and WT DCs following stimulation with a suilysin-deficient mutant strain used in previous studies [6], [10], [14]. No differences were observed between the parental and the suilysin-deficient strain (data not shown), excluding a major role for the suilysin in TLR4-mediated CXCL1 release.

NOD2^−/−^ DCs were also stimulated with *S. suis* parental strain and mutants. The release of IL-23 was significantly impaired in NOD2^−/−^ DCs stimulated with all *S. suis* strains tested (Figure 5A). The release of CXCL1 by NOD2^−/−^ DCs was also significantly reduced except when stimulated with the non-encapsulated strain (Figure 5B). No differences were observed between WT DCs and NOD2^−/−^ DCs in the release of other cytokines (results not shown).

### Non-redundant Activation of TLR2 and NOD2 Contributes to IL-23 Production by *S. suis*-stimulated DCs

It has been previously shown that IL-23 has an important role in bacterial infections and NOD2 activation seems to be highly responsible for DC elevated IL-23 production [31]. As in the case of *S. suis*, IL-23 was found to be TLR2- and NOD2-dependant, we investigated if blocking both pathways would further inhibit the release of this cytokine. NOD2^−/−^ DCs were pre-treated with a neutralizing antibody against TLR2. The efficiency and specificity of the neutralizing antibody was evaluated by stimulating DCs with the TLR2-ligand PAM(3)CSK(4) (data not shown). However, as shown in Figure 5C, there was no difference in the production of IL-23 by TLR2^−/−^ DCs, NOD2^−/−^ DCs and NOD2^−/−^ DCs pre-treated with the neutralizing antibody. The inhibition observed in either case was partial, compared to complete abrogation of IL-23 production in MyD88^−/−^ DCs. Hence, the release of IL-23 by *S. suis*-stimulated DCs might involve complex synergies between TLR2, NOD2 and other unknown TLRs. Similar results were obtained for CXCL1 (results not shown).

### Dual Deficiency in TLR2 and TLR9 Results in Significant Decrease in CD40 Expression and in IL-12p70 and CXCL10 Production

As mentioned above, the surface expression of CD40 was found to be MyD88-dependent, but TLR2-independent, suggesting a major role played by other TLR-dependent pathways. Since it has very recently been described a potential role of TLR9 in *S. suis* cell activation [32], the involvement of such receptor was investigated by pre-treating WT and TLR2^−/−^ DCs with ODN2088, an inhibitory oligonucleotide for TLR9 [29]. We first confirmed the neutralization specificity and efficacy of ODN2088 by inhibition studies of the TLR9-activator ODN1826 (data not shown). No differences in the expression of CD40 were noticeable with the single inhibition of TLR9. However, dual deficiencies in TLR2 and TLR9 resulted in a reduced expression of CD40 when DCs were stimulated with *S. suis* parental strain 31533 (Figure 6).

As the release of IL-12p70 and CXCL10 was also found to be TLR2-independent but MyD88-dependent, we also investigated the involvement of TLR9 in the release of IL-12p70 and CXCL10 by *S. suis*-stimulated DCs. No difference in the release of either cytokine was noticeable with the inhibition of TLR9 alone. However, dual deficiencies in TLR2 and TLR9 resulted in a significantly decreased release of both cytokines (Figure 7). Thus, these two receptors might act in a redundant or compensatory manner.

## Discussion

The mechanisms involved in the innate and adaptive immune responses toward *S. suis* remain essentially poorly known, and the increase in severity of *S. suis* infections in humans underscores the critical need of a better understanding of the interactions between *S. suis* and the immune system to generate an effective immune response against this pathogen. DCs are activated in the presence of *S. suis,* undergoing a maturation process characterized by the up-regulation of costimulatory molecules and the production of pro-inflammatory mediators [10], [22], [33]. In addition, *S. suis* was previously shown to possess several virulence factors able to modulate such DC functions, potentially leading to a diminished or ineffective host immune response [10], [22], [33]. In the present work, we attempted to further identify receptors involved in the innate immune recognition of *S. suis* serotype 2 by DCs. Murine cells were used since they have been shown to be a highly useful model for *S. suis* infections *in vivo* and *in vitro* [6], [12]. In addition, the availability of knock-out mice allows the study of the precise role of some of the receptors. Finally, *S. suis* interactions with murine, porcine and human DCs are similar [10], [22], [33].

The actual role of TLR signaling in bacterial phagocytosis is controversial [21]. It has been reported that activation of the TLR signaling pathways by bacteria regulates phagocytosis at multiple steps including internalization and phagosomes maturation [30], [34]. The absence of TLR2 somehow delayed *S. pneumoniae* phagocytosis and killing by neutrophils [35]. On the other hand, TLRs were shown not to play any significant role in phagocytosis of Group B *Streptococcus* (GBS) by macrophages [36]. There is only one study where the role of TLRs in phagocytosis of a bacterial pathogen (*Streptococcus pyogenes*) by DCs is reported [29], showing an absence of any role of TLRs in the internalization and killing of this pathogen. Results from the present study indicate that, similarly to *S. pyogenes*, TLRs do not seem to be involved in *S. suis* phagocytosis by DCs as being shown to be independent from signaling through MyD88. It should be note that the general phagocytosis rate of a well encapsulated *S. suis* serotype 2 is usually low [10], [22], [33].

TLR/MyD88 pathway was shown to be essential to host defense against several Gram-positive bacteria such as *Staphylococcus aureus*, *S. pneumoniae* and GBS [37]–[39]. Similar to what has been reported for *S. pyogenes* [29], *S. suis*-induced expression of CD40, MHC-II and CD86 is MyD88-dependant. The production of different cytokines and chemokines by MyD88^−/−^ DCs exposed to *S. suis* was also shown to be dramatically reduced or completely abrogated, hence confirming a central role for TLRs in DC activation by *S. suis*. The impaired expression of NF-κB in MyD88^−/−^ DCs further suggests a pivotal role of MyD88 signaling in DC activation and maturation by *S. suis*. These results are in agreement with a previous study showing that MyD88 is the major downstream mediator of TLR-dependent *S. suis*-induced cytokine production by macrophages [40].

The requirement for the MyD88 signaling pathway suggests that one or several TLRs are involved in DC activation and maturation by *S. suis*. However, MyD88-independent pathways would also be implicated, to a lesser extent, in the release of some mediators, such as CXCL10, and in the expression of CD86, which induction levels were only partially reduced in *S. suis*-infected MyD88^−/−^ DCs. It has been reported that MyD88 deficiency does not alter *Listeria monocytogenes*-induced co-stimulatory molecule up-regulation on DCs in vivo [41]. Since the MyD88-dependent pathway is used by all TLRs except TLR3 [42], a partial role of this receptor might be suggested. Transcription of TLR3 mRNA in brains of *S. suis* infected mice has been described [12]. In addition, a TLR4-mediated but MyD88-independent pathway has been reported to mediate LPS induction of CXCL10 via the TRIF/TRAM arm [43]. The MyD88-independent (TRIF/TRAM) pathway is also activated by TLR3 and is functionally responsible for activation of type I IFN and other IFN-inducible genes, such as CXCL10 [44]. Since TLR4 was not required for *S. suis*-induction of CD86 expression or CXCL10 release by DCs, a partial contribution of TLR3/TRIF pathway in *S. suis*-modulation of DC functions remains to be elucidated.

In order to further study TLRs implicated in the MyD88-dependent arm, DCs lacking TLR2 were infected with *S. suis*. Surface expression of CD86 and MHC-II, as well as the release of most mediators were found to be TLR2-dependent (but TLR4-independent), as previously suggested [40]. An implication of TLR2 and TLR6 in the recognition of *S. suis* by peripheral blood mononuclear cell (PBMC) and transfected epithelial cells was also reported [32], [45]. A study with swine DCs showed an up-regulation of relative expression of TLR2 and TLR6 mRNA after stimulation with *S. suis* [22]. Interestingly, the induction of three important mediators of T cell activation (CD40, IL-12p70 and CXCL10) was found to be TLR2-independent, which may indicate involvement of different TLR/MyD88 pathways. It has been previously described that TLR9 is also a receptor for the release of IL-12p70 [46]. TLR9 has recently been shown to be involved in *S. suis* activation of PBMC by either heat-killed bacteria or bacterial DNA [32]. In the present study, inhibition of TLR9 did not affect DC maturation and activation; however, deficiency in TLR2 and blocking of TLR9 together significantly affected the surface expression of CD40 as well as the production of both cytokines. A similar cooperation and/or redundancy between TLR2 and TLR9 was shown to be involved in splenic cytokine production by *S. pneumoniae* [47] and in activation of macrophages and DCs infected by *Mycobacterium tuberculosis* [48]. Finally, TLR4 does not seem to play an important role in DCs maturation and activation by *S. suis*. The suilysin, although highly related to the pneumolysin (originally reported to be recognized by this receptor [19]), would play a minor role in DCs activation. Interestingly, it has been recently reported that the pneumolysin can also activate DCs through a TLR4-independent pathway [49].

Another major finding of this work is the involvement of the cytosolic receptor NOD2 in the release of CXCL1 and IL-23 by DCs following stimulation with *S. suis*. IL-23 is a member of the IL-12 family, and is particularly efficient in supporting IFN-γ production and proliferation in memory T cells [50]. CXCL1 is one of the CXCL8 homologs believed to be important in the trafficking and activation of neutrophils in mice [51]. The involvement of NOD2 in cell responses to Gram positive pathogens, such as *S. pneumoniae*, *S. aureus* and *L. monocytogenes*, have also been described [52]–[56]. Since crosstalk and/or synergy between TLRs and NODs receptors have previously been proposed [57], [58], a possible interaction between TLR2 and NOD2 for *S. suis* DC activation was studied. Our results suggest that a complex non-redundant activation of both receptors seems to be involved in the release of CXCL1 and IL-23. Activation of a cytosolic receptor by a well encapsulated extracellular pathogen was not expected. Although in low numbers, some bacteria can be found inside DCs [10], [22], [33] which might, in theory, explain such activation. Exact mechanisms used by *S. suis* to activate NOD2 are so far unknown. Nevertheless, it has been proposed that cross-talks between cytosolic NODs and membrane-bound TLRs enhance responses to the multiple antigens simultaneously presented by a microbe [16], [59]. In addition, TLR2 activation has also been reported for some bacterial species to ensure digestion of bacterial cell wall and release of PG, which may activate NOD2 [60].

The presence of CPS in *S. suis* is known to hide cell wall antigens and thus reduce cell activation [6], [10], [22]. However, studies to date have identified cytokines for which the CPS is required for optimal induction, such as IL-1β [10], [13], [22], as also observed in this study. In the absence of CPS, uncovered cell wall components seem to activate DCs through multiple TLRs. However, modifications of cell wall components do not significantly change results of DC maturation and activation by *S. suis*. The presence of deacetylase genes in some pathogenic bacteria indicates that PG N-deacetylation could be a general mechanism used by bacteria to evade the host innate immune system [61]. Interestingly, in the case of *L. monocytogenes*, the N-deacetylation of PG allows the bacteria to avoid recognitions by NLRs, such as NOD [62]. This may be explained by the fact that the latter pathogen is usually found intracellularly. In the case of *S. suis*, cell wall modifications present in the double-mutant (PG/LTA) did not have any influence in modulation of DC activation by this receptor, probably due to the fact that relatively low number of intracellular bacteria are usually found, so low levels of PG are available to interact with NOD receptors.

This study confirms the hypothesis that recognition of *S. suis* by DCs seems to require a multimodal recognition system. Based on our results, a model of *S. suis* recognition by DCs is proposed (Figure 8). MyD88 signaling, mainly through TLR2, would be crucial for DC activation and maturation in response to *S. suis* infection. TLR9 (in conjunction with TLR2) and NOD2 were also involved in cell activation. However, other receptors, including other TLRs (such as TLR3), may mediate activation and maturation of DCs by *S. suis* and participate in the activation of the immune response. A role of NLRs, as recently described for GBS [63], cannot be ruled out. Further studies on these receptors are warranted.

## Supporting Information

Figure S1 **Surface expression of co-stimulatory molecules by DCs in response to** ***S. suis*** **.** WT and MyD88^−/−^ DCs (10^6^ cells/ml) were stimulated with S. *suis* WT strain 31533 (10^6^ CFU/ml) for 16 h. Non-stimulated cells served as negative control (C-). (A) Percentage of CD40 positive cells. (B) Percentage of CD86 positive cells. (C) Percentage of MHC-II positive cells. Twenty thousand gated events were acquired per sample. Quadrants were drawn based on FITC- and PE-control stains and were plotted on logarithmic scales. CD40, CD86 and MHC-II histograms were obtained by gating cells based on positive CD11c staining.
(TIF)

Figure S2 **CXCL1 production by DCs stimulated with suilysin-deficient** ***S. suis*** **mutant strain.** WT and TLR4^−/−^DCs (10^6^ cells/ml) were stimulated by different *S. suis* strains (10^6^ CFU/ml) for 16 h. Non-stimulated cells served as negative control (C-). Sample dilutions giving optical density readings in the linear portion of the ELISA standard curves were used to quantify cytokine levels. * P<0.05 denotes values that are significantly lower than those obtained with WT DCs.
(TIF)
