# Supplementary figures and images for: Immune Receptors Involved in Streptococcus suis Recognition by Dendritic Cells
Source: PLoS One. 2012 Sep 12;7(9):e44746. doi: 10.1371/journal.pone.0044746 (PMC3440357; doi:10.1371/journal.pone.0044746)

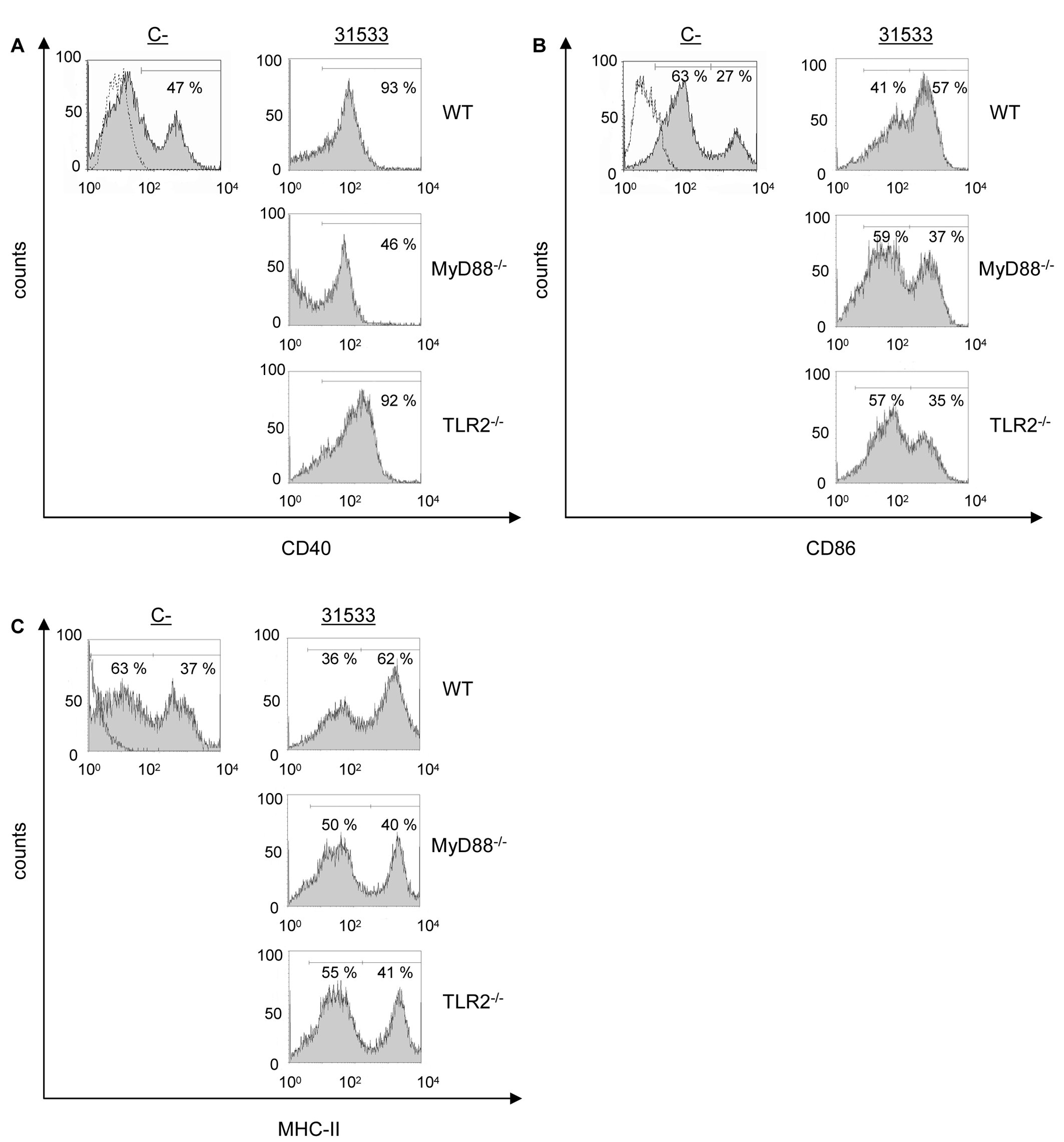

Supplement: Figure S1 — Surface expression of co-stimulatory molecules by DCs in response to S. suis . WT and MyD88−/− DCs (106 cells/ml) were stimulated with S. suis WT strain 31533 (106 CFU/ml) for 16 h. Non-stimulated cells served as negative control (C-). (A) Percentage of CD40 positive cells. (B) Percentage of CD86 positive cells. (C) Percentage of MHC-II positive cells. Twenty thousand gated events were acquired per sample. Quadrants were drawn based on FITC- and PE-control stains and were plotted on logarithmic scales. CD40, CD86 and MHC-II histograms were obtained by gating cells based on positive CD11c staining. (TIF) [file pone.0044746.s001.tif]

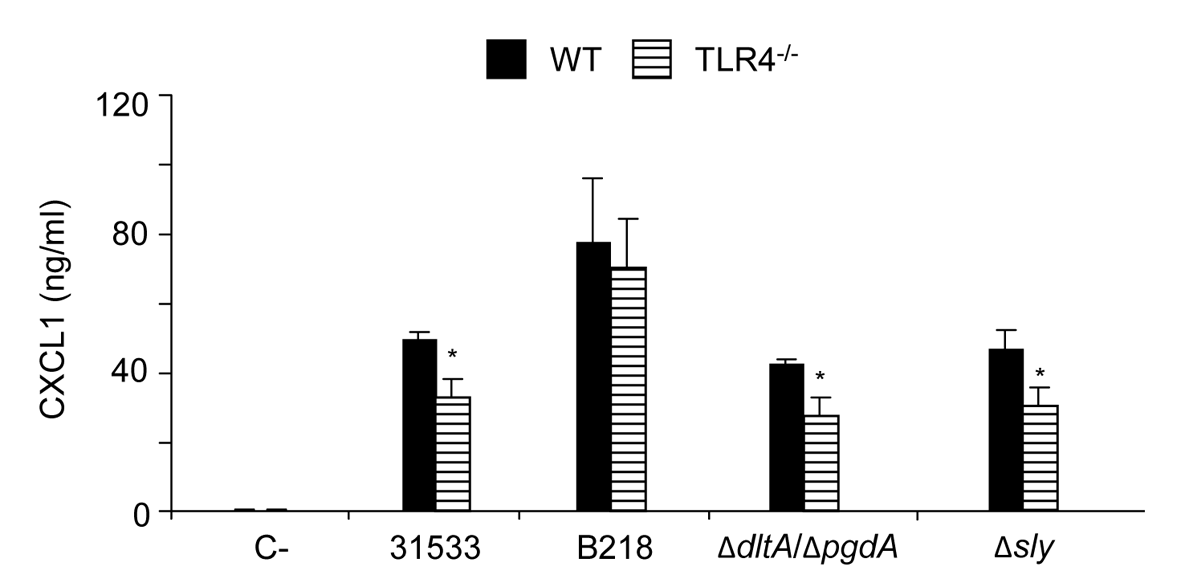

Supplement: Figure S2 — CXCL1 production by DCs stimulated with suilysin-deficient S. suis mutant strain. WT and TLR4−/−DCs (106 cells/ml) were stimulated by different S. suis strains (106 CFU/ml) for 16 h. Non-stimulated cells served as negative control (C-). Sample dilutions giving optical density readings in the linear portion of the ELISA standard curves were used to quantify cytokine levels. * P<0.05 denotes values that are significantly lower than those obtained with WT DCs. (TIF) [file pone.0044746.s002.tif]
